# The Prognostic Implications of the Geriatric Nutritional Risk Index in Patients with Prostate Cancer: A Single-Center Retrospective Cohort Study

**DOI:** 10.3390/healthcare13243266

**Published:** 2025-12-12

**Authors:** Rong Zhou, Yanqiong Zhou, Xiao Yue, Mei Wang, Yucong Zhang, Chang Liu

**Affiliations:** 1 Department of Geriatrics Medicine, Tongji Hospital, Tongji Medical College, Huazhong University of Science and Technology, Wuhan 430074, China; zhourong@tjh.tjmu.edu.cn (R.Z.); zhouyanqiong2025@163.com (Y.Z.); yxenable@tjh.tjmu.edu.cn (X.Y.); 1517062861@tjh.tjmu.edu.cn (M.W.); liuchangtjh@163.com (C.L.); 2 Department of Nursing, Tongji Hospital, Tongji Medical College, Huazhong University of Science and Technology, Wuhan 430074, China

**Keywords:** prostate cancer, aged, nutritional status, postoperative complications, hospital costs, length of stay

## Abstract

**Background and Aims:** Nutritional risk is a significant yet often overlooked factor influencing postoperative outcomes in older patients with prostate cancer. This study aimed to evaluate the impact of the Geriatric Nutritional Risk Index (GNRI) on postoperative complications in older patients undergoing radical prostatectomy. Secondary objectives included examining the association between the GNRI and healthcare resource utilization, specifically the length of hospital stay and hospitalization costs. **Methods:** This retrospective cohort study included patients aged ≥ 65 years who underwent laparoscopic radical prostatectomy at a single tertiary center between 2022 and 2024. Patients were stratified into a malnutrition group (GNRI ≤ 98) and a normal nutrition group (GNRI > 98). Clinical outcomes were compared using chi-square and t tests. Binary logistic regression was performed to identify independent predictors of complications, hospital stay, and costs. **Results:** Of the 264 patients included, 34.8% were classified as being at nutritional risk. The malnutrition group had a significantly higher incidence of postoperative complications (OR = 2.859, *p* = 0.007), longer hospital stays (OR = 4.678, *p* < 0.001), and greater hospitalization costs (OR = 4.867, *p* < 0.001). Nutritional risk remained a significant predictor after adjusting for confounders. **Conclusions:** GNRI-defined nutritional risk is independently associated with increased postoperative complications and higher healthcare resource utilization in older prostate cancer patients. The GNRI may serve as a practical and accessible tool for perioperative risk stratification in this population.

## 1. Introduction

Prostate cancer is the second most frequently diagnosed malignancy among men worldwide, with a median age at diagnosis of approximately 72 years [1,2]. Older patients undergoing surgical treatment are particularly vulnerable to postoperative complications—including delirium, infection, and deep vein thrombosis (DVT)—due to age-related declines in physiological reserve and comorbidity burden [3,4,5].

Nutritional status plays a crucial yet often underrecognized role in postoperative recovery in this population. Malnutrition, which may be aggravated by surgical stress or androgen deprivation therapy, has been associated with higher complication rates, prolonged hospitalization, and slower functional recovery in elderly patients undergoing urological procedures [6,7,8,9,10,11]. Similar findings have been reported in other surgical populations, where preoperative malnutrition was independently correlated with increased postoperative complications and delayed recovery [12,13]. Following laparoscopic radical prostatectomy (LRP), the rapid return to oral intake may give the false impression of adequate nutritional status, contributing to the under-screening of nutritional risk in clinical practice [14,15].

Several instruments, such as the Nutritional Risk Screening (NRS-2002) and *Mini* Nutritional Assessment (MNA), can be used for evaluating perioperative nutrition. Among them, the Geriatric Nutritional Risk Index (GNRI)—calculated from serum albumin and body weight—provides an objective, efficient, and widely validated tool for assessing nutritional risk in older adults, despite its sensitivity to inflammation and fluid shifts [16]. A recent meta-analysis involving more than 5500 cancer patients demonstrated that lower GNRI scores were significantly associated with postoperative complications (OR = 1.768, 95% CI: 1.445–2.163, *p* < 0.001), particularly in gastrointestinal malignancies [17]. Additional evidence indicates that the GNRI also holds prognostic value in elderly patients undergoing major oncologic procedures, such as hepatocellular carcinoma surgery [18]. However, its prognostic implications in prostate cancer surgery—especially regarding healthcare utilization such as length of stay and hospitalization costs—remain insufficiently explored.

To address this gap, the present study evaluates whether the GNRI is associated with postoperative complications and healthcare resource use among older patients undergoing radical prostatectomy. We proposed the following hypotheses:

(1) Lower GNRI scores are associated with a higher incidence of postoperative complications;
(2) GNRI-defined nutritional risk is independently associated with increased healthcare utilization, including prolonged hospital stay and higher hospitalization costs.

## 2. Materials and Methods

### 2.1. Patients

A consecutive cohort of patients aged ≥65 years who underwent laparoscopic radical prostatectomy (LRP) at a tertiary hospital in Hubei Province between August 2022 and August 2024 was included.

The inclusion criteria were as follows:

(1) Histologically confirmed prostate cancer;
(2) Localized disease (cT1–T2N0M0) on preoperative imaging;
(3) Completion of GNRI assessment prior to surgery.

The exclusion criteria included the following:

(1) Receipt of chemotherapy or radiotherapy within 3 months prior to surgery;
(2) Severe metabolic disorders (e.g., diabetic ketoacidosis, thyrotoxic crisis);
(3) Lower extremity edema or imaging evidence of effusion (pleural, peritoneal);
(4) Chronic gastrointestinal conditions (e.g., liver failure, Crohn’s disease);
(5) Missing >20% of required clinical data.

A total of 264 patients met the criteria and were included in the analysis.

**Figure 1:**
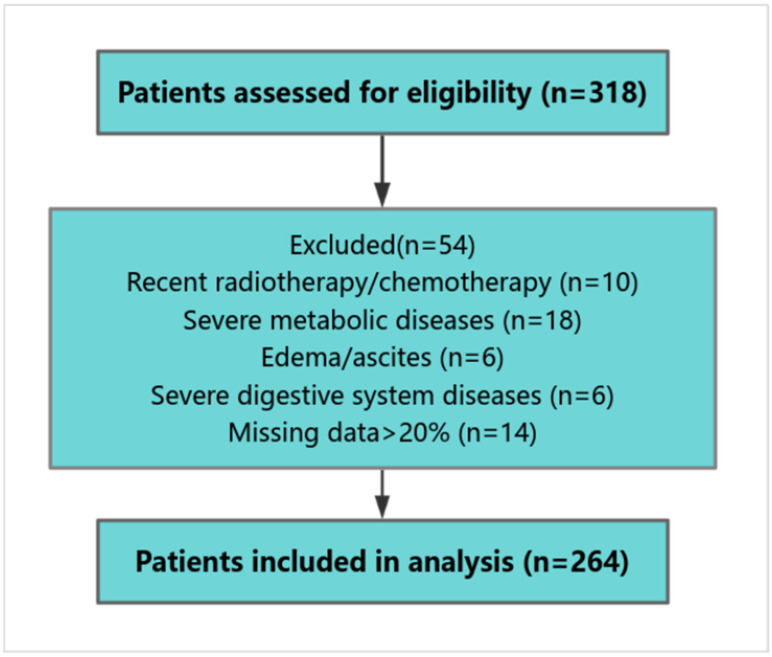
Flowchart of patient selection.

The patient selection process is summarized in Figure 1.

### 2.2. Clinical and Laboratory Data Collection

Data were extracted from the hospital information system (HIS) and follow-up database via the Yidu Cloud platform. Two independent investigators performed data extraction and cross-verification; discrepancies were resolved by a third reviewer to ensure data integrity.

Baseline variables included age, education level, sleep quality, polypharmacy, height, weight, comorbidities, smoking, alcohol use, prealbumin, white blood cell count, lymphocyte count, hemoglobin, platelets, ASA-PS score (ASA), and operation duration. Laboratory variables were categorized according to standard reference ranges. Sleep quality (from nursing records) was dichotomized as normal vs. abnormal (e.g., difficulty falling asleep, light sleep, frequent dreams, early waking). Polypharmacy was defined as concurrent use of ≥5 medications.

Outcomes:

- Primary outcomes: These outcomes included postoperative complications and health economic indicators. Postoperative complications—such as delirium, persistent fever ≥ 38.5 °C, lower-limb deep venous thrombosis, incomplete intestinal obstruction, and poor wound healing—were clinically diagnosed and documented in electronic medical records by attending physicians. These events were further verified based on routine clinical assessments, laboratory tests, and imaging examinations when applicable.

The Clavien–Dindo classification system was not applied because this study focused on nutritional risk-related complications and relied on retrospectively extracted diagnoses rather than intervention-based severity grading. No deaths occurred in this cohort.

- Economic outcomes: Length of hospital stay and direct inpatient costs (surgery, medications, diagnostics, nursing care) were assessed. Indirect costs (e.g., productivity loss, caregiving, rehabilitation) were excluded. Costs were recorded in Chinese Yuan (CNY); an approximate exchange rate of USD 1 ≈ CNY 7.2 was used for international reference. Due to the right-skewed distributions, both length of stay and costs were dichotomized using their medians. Scatter plots were generated to visualize the continuous relationship between the GNRI and these economic variables.

### 2.3. Definition of GNRI

Preoperative height, weight, and serum albumin were used to calculate the GNRI and BMI.

GNRI formula:

*GNRI =* 1.489 × *serum albumin* (g/L) + 41.7 × (*actual weight*/*ideal weight*)

Ideal weight formula (males):

*Ideal weight* (kg) = *height* (cm) − 100 − [(*height* − 150)/4.0]

A GNRI ≤ 98 indicated nutritional risk, while >98 indicated normal status, consistent with the thresholds validated by Sato et al. in high-risk prostate cancer cohorts [19]. Patients were classified as follows: MNg (malnutrition group): GNRI ≤ 98; NNg (normal nutrition group): GNRI > 98.

### 2.4. Statistical Analysis

Data were analyzed using SPSS 26.0.

Continuous variables were tested for normality. Normally distributed variables were compared using independent-sample t tests, while skewed variables were analyzed using the Mann–Whitney U test. Categorical variables were compared using chi-square tests. Multivariate logistic regression was performed to identify independent factors associated with postoperative complications, hospital stay, and hospitalization costs. Odds ratios (ORs) and 95% confidence intervals (CIs) were reported. A two-sided *p* value < 0.05 was considered statistically significant.

### 2.5. Ethics Approval

This study was conducted in accordance with the Declaration of Helsinki and was reviewed and approved by the Ethics Committee of Tongji Hospital, Tongji Medical College, Huazhong University of Science and Technology (Approval No. TJ-IRB202404014). As this was a retrospective study using de-identified patient data extracted from the “Yidu Cloud” database, the requirement for obtaining informed consent from individual patients was waived by the Ethics Committee.

## 3. Results

### 3.1. Differences in Baseline Characteristics Between the Two Patient Groups

A total of 264 patients undergoing LRP were included, with a mean age of 71.7 ± 4.5 years. Notably, 62.5% of the patients were classified as ASA III, and 121 patients (45.8%) had comorbidities. Additionally, patients in the MNg exhibited a significantly higher mean age (t = 5.002, *p* = 0.018) and lower levels of prealbumin (χ^2^ = 6.357, *p* = 0.012) and hemoglobin (χ^2^ = 7.274, *p* = 0.007). Furthermore, the proportion of patients with polypharmacy was significantly higher in the MNg (χ^2^ = 13.49, *p* = 0.001). No statistically significant differences were observed between the two groups in terms of education level, lifestyle factors (smoking and alcohol consumption), sleep status, presence of comorbidities, or surgical duration (*p* > 0.05 for all). Detailed results are presented in Table 1.

### 3.2. Differences in Postoperative Outcomes Between Patients with and Without Nutritional Risk

**Figure 2:**
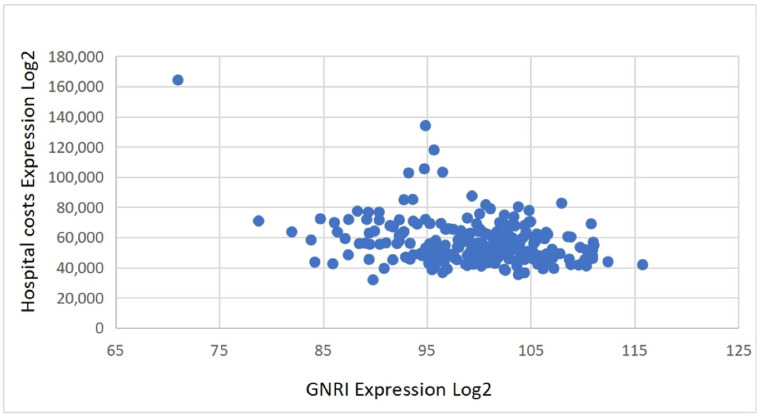
Correlation between GNRI and hospital costs.

**Figure 3:**
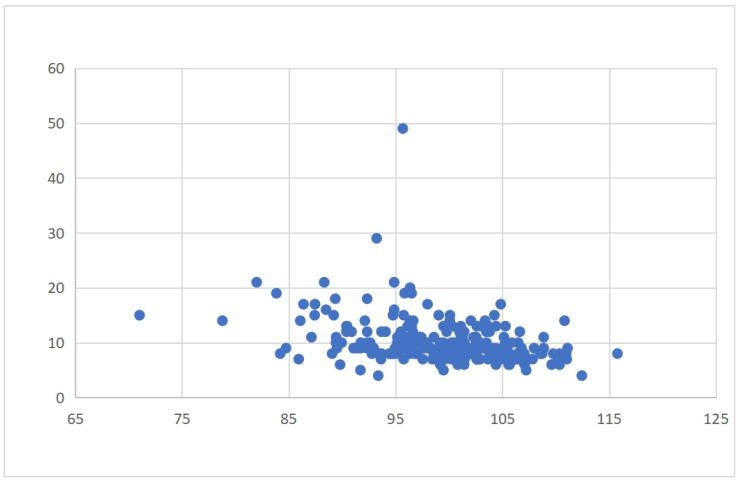
Correlation between GNRI and length of hospital stay.

Patients in the MNg experienced significantly worse clinical outcomes and greater healthcare resource use. Postoperative complications were more frequent in this group (*χ*^2^ = 27.65, *p* < 0.001), particularly for deep vein thrombosis (DVT) (*χ*^2^ = 17.875, *p* < 0.001). Hospital stays were also longer (*χ*^2^ = 32.1, *p* < 0.001) and hospitalization costs higher (*χ*^2^ = 41.882, *p* < 0.001) in the MNg. Table 2 and Table 3 summarize these outcomes. Scatter plots further confirmed that lower GNRI values tended to correlate with increased hospital stay durations and total costs, highlighting a continuous inverse relationship between nutritional status and healthcare resource consumption (Figure 2 and Figure 3).

### 3.3. Multivariate Regression Analysis of Postoperative Outcomes

Binary logistic regression revealed that nutritional risk (GNRI ≤ 98) was an independent predictor of all three outcome measures. Specifically, patients with nutritional risk had a significantly higher risk of postoperative complications (*OR* = 2.859, 95% *CI:* 1.335–6.121, *p* = 0.007), along with ASA III classification and comorbidities. Nutritional risk was also independently associated with prolonged hospital stay (*OR* = 4.678, 95% *CI:* 2.443–8.955, *p* < 0.001), after adjusting for comorbidities. Similarly, it predicted increased hospitalization costs (*OR* = 4.867, 95% *CI:* 2.547–9.301, *p* < 0.001), along with ASA III and comorbidities. These findings highlight the consistent prognostic value of nutritional risk across both clinical and economic outcomes (Table 4, Table 5 and Table 6). The negative association between GNRI and both hospitalization costs and length of stay is illustrated in Figure 2 and Figure 3 and described in the text for clarity.

## 4. Discussion

### 4.1. The Prevalence of Nutritional Risk Is High Among Older Patients with Prostate Cancer

In this study, the GNRI identified 34.8% of older prostate cancer patients as being at nutritional risk. Nutritional status was significantly associated with age, polypharmacy, prealbumin levels, and hemoglobin levels. Interestingly, although polypharmacy was more common among patients with nutritional risk, the number of documented comorbidities was paradoxically higher in the normal nutrition group. This pattern suggests that polypharmacy may capture hidden clinical vulnerability—such as functional decline, frailty, or subclinical conditions including sarcopenia and gastrointestinal dysfunction—rather than simply reflecting the number of diagnosed diseases. Frailer patients may also underreport comorbidities due to cognitive or physical limitations, contributing to this apparent discrepancy. These findings underscore the importance of integrating functional and nutritional indicators, in addition to comorbidity counts, into geriatric risk stratification.

Our results align with those of Shu et al. [20], who reported a 35.4% prevalence of nutritional risk among prostate cancer patients using the GNRI. As prostate cancer primarily affects men aged ≥ 70 years, nutritional vulnerability may be exacerbated by age-related factors such as tooth loss, impaired chewing and swallowing, and reduced digestive efficiency [21]. Hormonal alterations—including decreased growth hormone secretion and increased insulin resistance—promote protein catabolism and muscle loss, contributing to “sarcopenic obesity” [22]. In addition, tumor-related metabolic changes, such as enhanced glycolysis and protein breakdown, further deplete nutritional reserves [23].

Given that advanced genomic tests for prostate cancer remain costly and underutilized in many clinical settings [24], the GNRI provides a simple, accessible, and cost-effective alternative for assessing nutritional risk in older patients.

### 4.2. Mechanistic Links Between Nutritional Risk and Postoperative Complications

This study found that GNRI-defined nutritional risk was significantly associated with a higher incidence of postoperative complications. Patients in the malnutrition group had 2.86 times greater odds of developing complications compared with those with normal nutritional status (OR = 2.859, 95% CI: 1.335–6.121, *p* = 0.007), with notable increases in postoperative fever, delirium, and deep vein thrombosis (DVT). These findings suggest that the GNRI may serve as a practical tool for perioperative risk stratification. The elevated complication rates may be related to impaired immune function and reduced physiological resilience in malnourished patients.

Malnutrition can weaken humoral immunity by lowering immunoglobulin and complement protein production [25]. Although detailed immune profiling was not conducted, lymphocyte count was included as a surrogate indicator of immune status. Previous studies have shown that lymphocytopenia—common in malnourished individuals—may compromise cellular immunity, potentially contributing to the higher rate of postoperative fever observed in the malnutrition group (20.65% vs. 10.47%). Delirium occurred in 27.17% of malnourished patients, consistent with the findings in the study by Qian et al. [26], who reported that the GNRI was associated with postoperative delirium. Nutritional deficiencies, systemic inflammation, and metabolic stress likely contribute to neurocognitive vulnerability in this population [27].

DVT was also more frequent in the malnutrition group (11.96%), possibly due to sarcopenia-related immobility. Muscle weakness, prolonged bed rest, and reduced calf muscle pump function increase venous stasis and elevate thrombosis risk. The effects of malnutrition may be further amplified by common age-related comorbidities—such as cardiovascular disease, diabetes, and chronic kidney disease—which share overlapping inflammatory and metabolic pathways with malnutrition. These interactions may reduce physiological reserve and impair recovery. Thus, the GNRI may reflect not only nutritional status but also a broader cumulative health burden.

Although this study focused on urological surgery, comparable findings have been reported in other surgical contexts. Takano et al. [28] reported an association between the GNRI and infectious complications following gastrointestinal surgery, indicating that the clinical relevance of the GNRI may extend broadly across elderly surgical populations.

### 4.3. Nutritional Risk and Healthcare Resource Utilization

Older prostate cancer patients are at increased risk of prolonged hospitalization and higher medical costs due to reduced physiological reserves and complex comorbidities [29]. In this study, GNRI-defined nutritional risk was significantly associated with longer hospital stay (OR = 4.678, *p* < 0.001) and higher inpatient costs (OR = 4.867, *p* < 0.001) among patients undergoing laparoscopic radical prostatectomy (LRP). Hospitalization costs in our analysis included only direct medical expenses, such as surgery, medications, diagnostics, and nursing care. Indirect costs, including caregiver burden or post-discharge rehabilitation, were not captured and are acknowledged as a limitation.

Malnutrition may contribute to prolonged hospitalization through a cycle of complications and increased care needs. For example, DVT (11.96% in the MNg) requires close monitoring and anticoagulation; delirium (27.17%) necessitates neuropsychiatric management; and infection risk increases with length of stay, leading to broader antibiotic use and higher nursing workload. These factors collectively increase resource consumption. Our findings reflect this burden: the median hospitalization cost in the MNg was CNY 59,890 compared with CNY 48,927 in the NNg, with a median length of stay of 9 vs. 7 days (both *p* < 0.001). This corresponds to an average additional cost of CNY 10,963 per patient with nutritional risk.

Although we did not perform a formal cost-effectiveness analysis, previous studies suggest that preoperative nutritional support—typically costing CNY 1000–CNY 1500—may reduce postoperative complications and length of stay sufficiently to offset intervention costs [30,31]. Combining GNRI-based screening with targeted nutritional supplementation and resistance training has shown potential to improve clinical outcomes and reduce healthcare expenditures [32,33].

Taken together, these results indicate that incorporating GNRI screening into preoperative evaluation pathways may support a more cost-conscious and personalized care model for older surgical patients.

### 4.4. Clinical Implications and Implementation Strategies

Although this study did not include structured nutritional interventions, the observed association between GNRI-defined nutritional risk and adverse outcomes—such as postoperative complications, prolonged hospitalization, and higher medical costs—highlights the importance of early nutritional screening in older patients undergoing radical prostatectomy.

The GNRI is a simple and low-cost index that relies on routinely collected clinical parameters, including serum albumin and body weight. Because these measures are widely available, GNRI-based screening can be incorporated into standard preoperative assessments, even in settings without specialized nutritional or genomic testing [34].

To support clinical implementation, GNRI calculation could be integrated into electronic preoperative assessment tools to automatically flag patients at risk and prompt timely referral for nutritional evaluation [35]. In practice, early interventions may include nutrition counseling, protein-rich diets, or oral nutritional supplements [36,37]. These strategies are feasible and scalable, including in resource-limited environments.

Successful implementation of GNRI-based screening requires coordination across disciplines. Pilot programs or quality-improvement initiatives may help evaluate feasibility, staff engagement, and potential benefits for patient outcomes. Incorporating GNRI into perioperative checklists—alongside established surgical risk scores—may further support individualized decision-making and early intervention.

In summary, GNRI-based screening may offer a practical approach to enhancing perioperative risk stratification, strengthening nutritional care, and improving the efficiency of managing older patients undergoing radical prostatectomy.

### 4.5. Study Limitations and Future Directions

This study has several limitations. As a retrospective single-center analysis, the findings may be affected by selection bias and residual confounding. Although multivariate regression was performed, important variables—such as frailty status, performance status, and use of androgen deprivation therapy—were not available and may have influenced the results. The absence of matching or propensity score methods also limits causal interpretation.

The GNRI cutoff of ≤98 was adopted from previous studies and was not re-evaluated with ROC analysis in this cohort. External validation in other populations is needed. Standardized grading of complications (e.g., Clavien–Dindo classification) was not available because of the retrospective design. In addition, hospitalization costs reflected only direct medical expenses and did not include indirect costs, indicating the need for more comprehensive economic evaluations.

Prospective multicenter studies are needed to validate the prognostic value of GNRI in prostate cancer surgery, refine cutoff values, incorporate frailty assessments, and determine the effectiveness of preoperative nutritional interventions.

## 5. Conclusions

This retrospective study found that GNRI-defined nutritional risk was significantly associated with postoperative complications, longer hospital stay, and higher hospitalization costs in older prostate cancer patients undergoing laparoscopic radical prostatectomy. The GNRI may serve as a practical indicator to help identify patients at elevated risk and to inform perioperative nutritional assessment.

However, the single-center retrospective design introduces inherent limitations, including potential selection bias and unmeasured confounders such as frailty, performance status, and androgen deprivation therapy. Although multivariate analysis was performed to adjust for baseline differences, the absence of matching or propensity score methods further limits causal interpretation. In addition, the GNRI cutoff of ≤98 was derived from previous studies from the literature and was not recalibrated using ROC analysis in this cohort. Future prospective multicenter studies are warranted to refine optimal GNRI thresholds and to incorporate standardized complication grading systems such as the Clavien–Dindo classification.

As genomic-based assessment tools remain limited in older patients due to costs and accessibility, the GNRI provides a simple and low-cost alternative for preoperative risk stratification. Our findings support the potential value of integrating GNRI-based nutritional screening into routine perioperative evaluation in geriatric oncology practice.

## Figures and Tables

**Table 1:** Results of the test of differences between the two groups of patients in terms of general information.

Variable	Category	Frequency/ Mean (Total)	Nutritional Risk		*χ*^2^/*t*	*p*
			NNg (n = 172)	MNg (n = 92)		
Age (years)		71.7 ± 4.5	70.8 ± 4.0	73.6 ± 4.8	5.002 b	0.018
Education	Primary school and below	97	62	35	0.108 a	0.948
Smoking	High school	109	72	37		
	University degree	58	38	20		
	No	172	112	60	0.000 a	0.987
	Yes	92	60	32		
Alcohol consumption	No Yes	202 62	130 42	72 20	0.239 a	0.625
Sleep	Normal Abnormal	171 93	117 55	54 38	2.286 a	0.131
Comorbidity	No Yes	143 121	95 77	48 44	0.226 a	0.635
Polypharmacy	No Yes	213 51	150 22	63 29	13.49 a	0.001
ASA	II	99	76	23	2.278 a	0.131
	III	165	96	69		
	<150	6	1	5		
Prealbumin (mg/L)	≥150	258	171	87	6.357 a	0.012
Hb (g)	<120 ≥120	39 225	18 154	21 71	7.274 a	0.007
White blood cell count	<3.5	8	4	4		
(×10^9^ g/L)	3.5–9.5	208	137	71	0.862 a	0.650
	>9.5	48	31	17		
Lymphocyte count	<1.1	71	41	30		
	1.1–3.2	189	129	60	2.920 a	0.232
(×10^9^ g/L)	>3.2	2	2	2		
	<125	13	6	7		
Platelet count (×10^9^ g/L)	125–135 >135	13 238	8 158	5 80	2.301 a	0.316
Operation duration (min)		143.36 ± 10.99	142.86 ± 11.17	144.30 ± 10.63	−1.017 b	0.310

Note: a indicates *χ*^2^ test, b indicates *t* test. MNg: Malnutrition group (GNRI ≤ 98). NNg: Normal nutrition group (GNRI > 98).

**Table 2:** Comparison of postoperative outcomes between the two patient groups.

Outcome Variable	Category	Nutritional Risk		*χ* ^2^	*p*
		NNg (n = 172)	MNg (n = 92)		
Postoperative complications	NO YES	138 34	45 47	27.65	<0.001
Hospital stays (days)	<9 ≥9	96 76	18 74	32.10	<0.001
Hospitalization costs (CNY)	≤52,361.5 >52,361.5	113 59	22 70	41.882	<0.001

**Table 3:** Comparison of the occurrence of postoperative complications between the two groups of patients.

Group	Postoperative Complications				
	Delirium	DVT	T ≥ 38.5 °C	Incomplete Intestinal Obstruction	Poor Wound Healing
MNg (n = 92)	25 (27.17%)	11 (11.96%)	19 (20.65%)	7 (7.61%)	5 (5.43%)
NNg (n = 172)	29 (16.9%)	1 (0.58%)	18 (10.47%)	4 (2.33%)	2 (1.16%)
*χ* ^2^	3.918	17.875	5.162	4.190	4.238
*p*	=0.048	<0.001	=0.023	=0.041	=0.040

**Table 4:** Multivariate regression analysis results for postoperative complications.

Variable	Category	*B*	*S.E*	*p*	*OR*	95% CI
Comorbidity ASA	No Yes II III	1.382 3.404	0.420 0.667	0.001 <0.001	1 3.982 1 30.086	1.746~9.078 8.138~111.225
Hb (g)	<150 ≥150	−1.176	0.529	0.026	1 0.308	0.109~0.871
Nutritional risk	NO YES	1.050	0.388	0.007	1 2.859	1.335~6.121

Adjusted variables included age, comorbidity, ASA classification, sleep disturbance, polypharmacy, education level, nutritional risk, and hemoglobin.

**Table 5:** Multivariate regression analysis results for length of hospital stay.

Variable	Category	*B*	*S.E*	*p*	*OR*	95% CI
Nutritional risk	NO YES	1.543	0.331	<0.001	1 4.678	2.443~8.955
Comorbidity	NO Yes	0.649	0.315	0.040	1 1.914	1.032~3.551

Adjusted variables included age, comorbidity, ASA classification, polypharmacy, prealbumin, and nutritional risk.

**Table 6:** Multivariate regression analysis results for hospitalization costs.

Variable	Category	*B*	*S.E*	*p*	*OR*	95% CI
Comorbidity	NO Yes	1.111	0.330	0.001	1 3.037	1.590~5.800
Nutritional risk	NO YES	1.583	0.330	<0.001	1 4.867	2.547~9.301
ASA	II III	1.217	0.299	<0.001	1 3.377	1.878~6.073

Adjusted variables included age, comorbidity, ASA classification, polypharmacy, prealbumin, and nutritional risk.

## Data Availability

Due to privacy or ethical restrictions, the datasets generated and analyzed during the current study are available from the corresponding author upon reasonable request.
